# Autobiographical memory and mentalizing impairment in personality disorders and schizophrenia: clinical and research implications

**DOI:** 10.3389/fpsyg.2012.00529

**Published:** 2012-11-26

**Authors:** Giancarlo Dimaggio, Giampaolo Salvatore, Raffaele Popolo, Paul H. Lysaker

**Affiliations:** ^1^Center for Metacognitive Interpersonal TherapyRome, Italy; ^2^School for Cognitive Therapy “Istituto A.T. Beck,”Rome, Italy; ^3^Scuola di Psicoterapia “Studi Cognitivi,”Milan, Italy; ^4^Roudebush VA Medical Center, Indiana University School of MedicineIndianapolis, IN, USA

Clinicians in the fields of mental health, neuroscientists, and social psychologists have been increasingly interested in how persons with psychiatric conditions experience a range of difficulties related to how they think about themselves and others. These difficulties include problems forming and retrieving the specific autobiographical memories (AM) that ground a sense of personal identity (McAdams, 2001). They have also involved diminishments in the capacity to engage in acts of mentalization or metacognition which allow for the understanding of mental states. Both of these forms of dysfunction are of increasing relevance given that each has been commonly observed across different psychiatric conditions, appears relatively independently from symptoms, and is uniquely linked to functional impairment (Dimaggio and Lysaker, 2010; Liu et al., in press).

To date deficits in AM and metacognition have mostly been analyzed separately. Psychopathology research has tended to explore either one or the other, thus neglecting the larger issue of how metacognition and AM are produced by systems which are likely closely related and likely feed and support one another (Markowitsch and Staniloiu, 2011). In development, AM and Theory of Mind (ToM) tend be influenced by common factors (Nelson and Fivush, 2004). Persons who present with overgeneralized AM also experience mentalizing problems including reduced affect awareness and poorer ToM (Palmieri et al., 2012).

Psychotherapy research and general neuroscience have begun to examine how these functions support one another and how disruptions in either AM or metacognition might lead to decrements in the other. To explore this issue we review psychopathology research concerned separately with problems in AM and metacognition in two forms of mental illness: schizophrenia and personality disorders (PD). We then discuss the advances from psychotherapy research and neuroscience for understanding how these two phenomena are related and comment on opportunities offered by these insights for scientific and clinical work.

## Autobiographical memories in persons diagnosed with psychiatric disorders

AM are understood to be a core aspect of healthy function across the human life span (Conway, 2005; Singer et al., in press). Being able to retrieve personal memories allows persons to form representations of themselves as unique beings who exist with meaningful continuity over time. They also provide a context for making sense of what is happening interpersonally in the moment.

Such processes often seem disrupted in persons suffering with significant forms of psychopathology. Instead of resorting to key past events as a prototype for meaning-making and action predictions, patients might resort to overgeneralized memories or intellectualizations, in order to make sense of their choices, dilemmas and conflicts. For instance, patients with Narcissistic PD might respond to an interpersonal conflict not by recalling specific memories about their live in which the conflict was solved, but rather with a generalization about others as inept or ungrateful. Patients diagnosed with psychosis might respond to the same dilemma without any clear recollection from the past, or if the past was recalled it might be disjointed and not clearly related to the problem in the moment.

To date, qualitative and quantitative work has suggested at least six different though related ways (Dimaggio and Semerari, 2001; Lysaker et al., 2001) in which AM are disturbed in psychosis and PD. First, AM in persons with these conditions often lack clear space and time boundaries. It is difficult to detect where and when an event has taken place. Second, when memories are retrieved they are often made sense of through the use of intellectualization and moral rules, without a nuanced sense of what actually happened between the specific people involved in the memory; for instance it might be recalled that certain people were ungrateful or demanding without there being sufficient detail to determine what might have led up to that event. Third, descriptions of others present in the narrative episode are often sketchy and the sequences of the actions and reactions are difficult to follow and bereft of detail.

Fourth, within the narrative episode the dialogue that takes place among the characters is often repetitive and stereotyped. Exchanges between people may be recalled but they tend to follow more of a formula than to reflect recollections of a unique conversation. Fifth, the narrative theme of the memory may be redundant, often reflective of the most basic or gross set of motives, and the story itself tends to be formulaic; memories may center around threat and fight/flight responses and end without any resolution or an irreversible resolution (e.g., a severing of connection to another person). A sixth problem is that AM recollected lack a pictorial quality which might enable a listener to imagine what happened. Memories may lack details related to visual, auditory, olfactory, or gustatory cues, elements which would lend an opportunity to understand the memory as a unique experience.

Turning to explorations of specific disorders, research has suggested that many patients with avoidant, dependent and obsessive-compulsive PD featured reduced AM specificity (Spinhoven et al., 2009). Similarly, narratives of patients with borderline PD have frequently been found to be overly general (Maurex et al., 2010), less often concerned with prototypical life events and moreover less coherent (Jørgensen et al., 2012). Autobiographical narratives produced by patients with borderline PD portray life events as either imbued with dysregulated affect and lacking any sense of agency (Adler et al., 2012).

Regarding schizophrenia, fundamental disruptions have been observed (Lysaker and Lysaker, 2008). Many with schizophrenia are less able to spontaneously recall memories which involved discrete events that occurred in a specific time and place (Corcoran and Frith, 2003; Riutort et al., 2003; D'Argembeau et al., 2008). There may be lesser recall of specific or well-defined memories, and a tendency to recall more public than private events (Cuervo-Lombard et al., 2007; Raffard et al., 2010). Further, the specific AM of patients with schizophrenia are less consistent with the overall self-image they endorse and the self lacks agency; this suggests a deficit not just in retrieving memories but also in integrating different aspects of identity into a coherent whole (Bennouna-Greene et al., 2012).

## Metacognitive/mentalizing dysfunctions in personality disorders and schizophrenia

Literature also suggests that patients with PD and schizophrenia suffer from a wide array of dysfunctions in thinking about themselves and others. Referred to as dysfunction in metacognition or mentalizing, this includes difficulties recognizing mental states, correctly naming them and using them in a flexible way and as a reliable source of information.

Research has specifically suggested that many with PD experience difficulties describing their own emotions, seeing links between emotion and thoughts (Semerari et al., 2007; Nicolò et al., 2011), and seeing their own thought processes in a detached and reflective way (Bateman and Fonagy, 2004; Semerari et al., 2005; Dimaggio et al., 2009a,b). Difficulties appraising the mental states of others has also been noted (Fonagy et al., 2002) though relatively less impairment has been observed in more basic laboratory ToM tasks (Domes et al., 2009; Ghiassi et al., 2010). The combination of poor mentalizing and PD have also predicted poorer vocational outcome (Bly et al., 2012); poorer mentalizing skills in patients with avoidant and borderline PD is a negative predictor of psychotherapy outcome (Gullestad et al., in press). In general, persons with PD appear to have limited abilities to use mentalization in order to respond to psychological problems (Carcione et al., 2011). The ability to reading the mind of the others within tasks that call for empathy have been found to be problematic for persons with narcissistic PD, and a deficit of which they are unaware of (Ritter et al., 2011).

More pronounced metacognitive deficits have been found in schizophrenia. Compared with persons without psychosis, deficits have been noted in a broad array of assessments of both cognitive and affective ToM tasks (Brüne, 2005; Penn et al., 2008), and these problems are associated with negative symptoms and poor social functioning (Brüne et al., 2011). The narratives of persons with schizophrenia feature severe inabilities to identify mental states, to see the world from multiple perspectives, and to use that knowledge to respond to psychological challenges (Lysaker et al., 2007; Mitchell et al., 2012) both as a trait marker (Lysaker et al., 2011b) and as a result of trauma (Lysaker et al., 2011c). Empathy has been found a problem for many with schizophrenia (Derntl et al., 2009).

## Neuroscience and the link of autobiographical memory with metacognition

In parallel with these developments, neuroscientists have also offered evidence of a close relationship between AM and metacognition. For instance imaging studies have suggested that AM is supported with brain areas which partially overlap with ones devoted to attributions to mental states (Rabin et al., 2010; Spreng and Grady, 2010; Mar, 2011; Rabin and Rosenbaum, 2012). Spreng and Mar (2012) have noted that even non-overlapping cortical areas linked with AM and mentalistic attributions, share a network of connections. Whitehead et al. (2009) have suggested that the ability to engage in pretend play is coupled with activations in cortical areas implicated in both ToM and narrative processes.

## The interplay of impairments in AM and metacognition in psychotherapy

While much psychopathology research has considered impairment in AM and metacognition separately, recent work in psychotherapy has begun to urge that successful treatment requires a consideration of the relationship of the two. Among those are mentalization-based treatment (Bateman and Fonagy, 2004), metacognitive interpersonal therapy for PD (Dimaggio et al., 2007, 2012), and metacognition-oriented psychotherapy for psychosis (Lysaker et al., 2007, 2011a; Buck and Lysaker, 2009; Salvatore et al., 2009, 2012). These approaches share the idea that a key aspect of therapy is to help patients to narrate specific AM—instead of resorting to overgeneralized memories or intellectualizations—as specific AM are the most fertile soil to think about for mental states (Dimaggio et al., 2010, 2012; Lysaker et al., 2011a). In other words, the enrichment of AM may promote improvements in metacognitive capacity. To address this possibility these procedures train therapists to seek specific memories. For instance, overgeneralized recalls have been suggested as a cue to explore for the specifics of narrative episodes (Dimaggio et al., 2012).

The rationale for seeking and thinking about specific episodes is based on the assumption that metacognitive reflection is at its heart a consideration of real life events, and as such impoverished AM offer little fertile ground for metacognitive activity. Accordingly, for many patients the appearance of more nuanced AM precedes the development of the ability to think in more complex ways about oneself and others; narrative episodes may provide opportunities for patients to recognize actual chains of thoughts, affects and behaviors in interpersonal interactions (Dimaggio et al., 2012). Other patients though may need to reach some basic level of access to mental states, in particular awareness of affects and their links with thoughts and behaviors, before their AM emerge. Formal analysis of case studies has shown that successful therapy involves the progressive growth of narrative capacity and aspects of metacognition, with the ability to narrate one's own story improving first, then followed by awareness of one's own states and awareness of the mental states of the others (Lysaker et al., 2005, 2007).

## Implications for treatment and research in psychopathology

While difficulties in AM and metacognition have been treated separately as factors which are present and influence outcome in significant forms of mental illness, psychotherapy, and neuroscience research suggests the need for these phenomena to be studied together. More efforts would seem to have the potential to answer several questions, including whether one form of deficit emerges before the other. Do metacognitive dysfunctions result in impoverished AM or diminished AM leave persons less able to think about mental states (Dimaggio et al., 2008, 2009a,b)? Are AM and metacognition best thought of as two networks operating together or a reflection of a single core psychological process? Are they decoupled in a similar way in non-clinical subjects and in different forms of psychopathology?

There is reason to believe that narrative enrichment (Gonçalves et al., 2012) and promoting mentalization (Bateman and Fonagy, 2009) are features of successful therapies; research addressing their interplay may guide the development of these interventions.
